# Empirical advances with text mining of electronic health records

**DOI:** 10.1186/s12911-017-0519-0

**Published:** 2017-08-22

**Authors:** T. Delespierre, P. Denormandie, A. Bar-Hen, L. Josseran

**Affiliations:** 1 Institut du Bien Vieillir Korian, 21-25 rue Balzac, 75008 Paris, France; 2 Research lab: EA 4047, UFR des Sciences de la Santé Simone Veil, UVSQ Université Paris-Saclay, 2 Avenue de la Source de la Bièvre, Montigny le Bretonneux, 78180 France; 3 MNH Group, 185 rue de Bercy, 75012 Paris, France; 4 UFR de Mathématiques et Informatique, Université de Paris Descartes, 45 rue des Saints-Pères, Paris, 75006 France

**Keywords:** Nursing homes, SQL query, Information extraction, Named entity recognition, Data mining, Text mining, Word cloud, Multiple component analysis, Principal component analysis, Hierarchical clustering

## Abstract

**Background:**

Korian is a private group specializing in medical accommodations for elderly and dependent people. A professional data warehouse (DWH) established in 2010 hosts all of the residents’ data. Inside this information system (IS), clinical narratives (CNs) were used only by medical staff as a residents’ care linking tool.

The objective of this study was to show that, through qualitative and quantitative textual analysis of a relatively small physiotherapy and well-defined CN sample, it was possible to build a physiotherapy corpus and, through this process, generate a new body of knowledge by adding relevant information to describe the residents’ care and lives.

**Methods:**

Meaningful words were extracted through Standard Query Language (SQL) with the LIKE function and wildcards to perform pattern matching, followed by text mining and a word cloud using R® packages. Another step involved principal components and multiple correspondence analyses, plus clustering on the same residents’ sample as well as on other health data using a health model measuring the residents’ care level needs.

**Results:**

By combining these techniques, physiotherapy treatments could be characterized by a list of constructed keywords, and the residents’ health characteristics were built. Feeding defects or health outlier groups could be detected, physiotherapy residents’ data and their health data were matched, and differences in health situations showed qualitative and quantitative differences in physiotherapy narratives.

**Conclusions:**

This textual experiment using a textual process in two stages showed that text mining and data mining techniques provide convenient tools to improve residents’ health and quality of care by adding new, simple, useable data to the electronic health record (EHR). When used with a normalized physiotherapy problem list, text mining through information extraction (IE), named entity recognition (NER) and data mining (DM) can provide a real advantage to describe health care, adding new medical material and helping to integrate the EHR system into the health staff work environment.

**Electronic supplementary material:**

The online version of this article (doi:10.1186/s12911-017-0519-0) contains supplementary material, which is available to authorized users.

## Background

Issues with nursing care narrative (CN) analysis [1], as well as with electronic health record (EHR) analysis [2–4], are recurrent, but data warehousing (DWH) [5] and cloud computing developments on the one hand [6] and data extraction techniques on the other hand [7, 8] have changed the way CN analysis is performed and used. Today, EHR is a valuable source of clinical information [4, 9], but the abundance of unstructured textual data in EHR presents a real challenge to realizing its full potential [8, 10, 11]. EHR, with the parallel rapid growth of CN, plus the need for improved quality of care and reduced medical errors, is a strong incentive for the development of natural language processing (NLP) [8]. The free text of the CN is a rich resource, in which health staff record events or information history as told to them by their patients [12] or the care provided linked to residents’ health status. However, working with this textual material depends strongly on the availability of NLP tools and expertise in using them. Much of the available clinical data from DWH are in narrative form and can be used as a convenient tool by health providers and medical staff. Thus, textual data are used most of the time through a professional data frame as a networking tool between health care professionals and not as a pertinent, therapeutic decision-making tool.

The (Korian) group specializes in medical nursing homes and acute and subacute care clinics. Since 2010, this European group has had health records with data coming from both the medical and social dimensions in four countries (France, Germany, Italy and Belgium), built through several professional DWHs using ORACLE® technology [13]. For every new resident admitted to one of its nursing homes (NHs), a personal electronic resident medical file is opened to collect individual patient data at different time points of the stay: at admission (admission date, medical history, marital status, birthdate, tastes and habits), on a daily basis (new pathologies, chronic disease evolution, date of death, drug prescriptions), or after specific medical or health care professional visits. Whereas data are recorded by different professionals following the resident’s situation, through structured, layered and indexed data organized through a hierarchically data scheme, CNs are mostly captured along the way in a few tables and are used extensively as networking tools. While these textual data include direct speeches, acronyms or simply words, could they actually contain relevant and reusable health information?

The objective of this study was therefore twofold: first, to illustrate how CN text mining processes could enhance electronic medical record (EMR) data; and then, to demonstrate the convergence of information between CN-extracted data and EMR data strictly speaking.

Physiotherapy care data were chosen for two main reasons: first, as a meaningful tool preserving motor function in frail, elderly people and preventing them from a long list of physical ailments, second as well-defined interventions directed to an identifiable target population inside the NH population [14]. The adjusted size and the well-defined sample allowed checking every step of the process, opening the way towards syndrome labelling, patient stratification and improved targeting of care [15].

## Methods

### Aim, design and settings

**Fig. 1:**
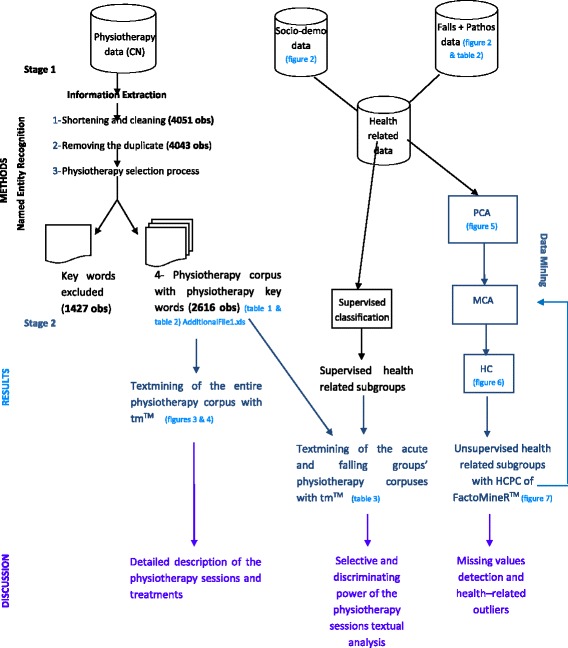
The experiment design with monitored (textual SQL and classification) and unsupervised (PCA, MCA, HC and textmining) techniques

Our goal was to show through this whole textual experiment (see Fig. 1) that text mining through IE, NER and DM techniques [8] could be essential to better follow residents’ health paths and improve their quality of care by adding new, simple, useable data, as well as valuable and matching information with the already existing EHR data.

The design to extract textual data from physiotherapy care narratives involved two information systems: the DWH, using ORACLE® queries to extract data from tables and build the physiotherapy corpus; and R Studio® for the statistics and data mining [16], as well as the textual analysis of the corpus (in black and dark blue, respectively, in Fig. 1).

### Characteristics of participants and material

The data source for the corpus analysis of clinical text was built through the selection of all of the residents alive on September 30, 2013 with at least one physiotherapy narrative during the previous 6 months [17]. This corpus contained a total of 4051 physiotherapy CNs for 1015 residents from 127 nursing homes located in eleven regions of France during the period from 04/01/2013 to 09/30/2013. These records were extracted from one table and de-identified and anonymized; see Additional file 1: Annex 1 for the physiotherapy corpus and Additional file 2: Annex 2 for the anonymization process [18–20]. Physiotherapy keywords and the most frequent corpus expressions were translated to generate all of the tables and Figs. 3 and 4. For full details of this process, see Additional file 3: Annex 3.

Data were also gathered from other tables (right side of Fig. 1): socio-demographic data with the residents’ ages and sexes, medical histories and pathologies on September 30, 2013, as well as their falling histories from the beginning of their NH stay through September 30, 2013; on the building of the 1015 anonymized residents’ health tables, see Additional file 4b: Annex 4.

In this sample, medical histories, pathologies and fall variables were cast from multiple event variables to synthetic counting variables to have only one record per resident (see Table 2).

### Description of the processes and statistical analysis

#### Building the physiotherapy variables: The textual SQL plus text mining strategy

The physiotherapy comments could take different formats (date, one or two words, sometimes even dull words, such as ‘*others’* or *No*, pre-constructed small sentences or free text) depending on the professional feeding the system. By analysing several hundreds of them through the two main stages described in Fig. 1, a list of physio keywords was built, where most of the time, one physio keyword stood for one physio concept.

By combining precisely monitored data mining techniques: designing specific physiotherapy textual SQL queries [21] and then going through unsupervised queries with text mining [22], a precise and easy-to-check textual description of the physiotherapy treatments was built with relevant physiotherapy variables.

The first stage was done on the DWH’s side and is detailed on the workflow Fig. 1:

1. shortening the character fields from 4000 to 300 and removing accentuation, dates or words such as *No*;
2. removing some overlapping CN;
3. excluding meaningless words or expressions CN using ORACLE® queries with the SQL LIKE function and wildcards to perform pattern matching [21];
4. building physiotherapy variables with the same technique to detect some keywords, such as ‘*walk’*, ‘*balanc*e’ or ‘*autonomy’* (see Table 1), describing physiotherapy care and counting them.

**Table 1:** The physiotherapy corpus built through the SQL process (stage 1) with selected one physio expression--one physio concept and their precision

word	frequency	percentage	Precision a priori	Sentence precision	Precision a posteriori
*free_text*	*707*	*17.45*	*N/A*	*N/A*	*N/A*
autonomy	560(524)	13.82	93.57	80.56	98.75
renewal	518	12.79	100	-	100
per_week	476	11.75	100	-	100
good	383(327)	9.45	85.37	94.64	99.22
functional_recovery	358	8.84	100	-	100
*non_classified*	*318*	*7.85*	*N/A*	*N/A*	*N/A*
walking	302	8	-	100	100
partial	295(294)	7.28	100	100	100
antalgic	222(208)	5.43	100	100	100
per_day	209	5.16	100	-	100
others	194	4.79	100	-	100
pain	86	1.88	-	96.51	96.51
balance	84	2.07	-	98.81	98.81
massage	74	1.83	-	100	100
participation	51	1.26	-	96.8	96.8
voluntary	47	1.16	-	100	100
motivation	37	0.91	-	100	100
stimulation	33	0.81	-	100	100
stopping_treatment	21	0.51	100	-	100
cognition	15	0.37	-	100	100
useless	11	0.27	100	-	100
modification_treatment	9	0.22	100	-	100

*N/A* not applicable

Every CN with one of these words was counted as one word occurrence. These words were selected after extensively checking and listing the CNs and iteratively checking the results. Among these built-in physiotherapy variables, we met three different situations:

a- one word-one concept always alone for words such as *‘useless’*;
b- one word-one concept alone most of the time and sometimes combined in sentences for words such as ‘*partial’*, *‘antalgic’, ‘good’, ‘functional recovery’* and*’ autonomy maintenance’*;
c- one word-one concept always combined in sentences for words such as *‘massage’, ‘pain’, ‘cognition’, ‘balance’* and *‘walk-walking’*.

Through this process, we could analyse the also defined observations’ lengths greater than or equal to 30 as those belonging to the free text class, using a cut-off from the lengthiest of our classified words and expressions (26 characters in French), and the non-classified class as those not containing any of the keywords listed in Table 1. These two classes as tools helped us to count and check all the meaningful words. Finally, for the physiotherapy variables described in cases *3* and *a* above, the precision of the one word-one concept was obviously of 100%. Then, for cases such as *b*, there was a precision a priori defined as the alone/together ratio, when the word-concept is used alone versus when it is used in sentences. In the other cases, *c* and *b in sentences* it had to be computed (two last columns in Table 1). The precision a posteriori aggregated the a priori and sentence precisions whenever applicable.

The second stage was conducted with RStudio®: all the non-classified CNs from the first phase (*non_classified* in Table 1) or the most frequent expressions describing the physiotherapy care found in the first stage, here *‘walking’*, *‘autonomy’* or *‘functional recovery’*, were aggregated in a single corpus and analysed through text mining. This technique based on stemming and lemmatization (combining words of the same family in the same group) used the R® package tm® [22].

After listing the words in a bar plot with the tm® R package (Fig. 3), the same corpus was analysed with three other R® packages -- SnowballC® [23], wordcloud® [24] and RColorBrewer® [25] -- to build a word cloud (Fig. 4) as a method of showing what comes at the forefront in the physiotherapist‘s concerns. The first package uses the C libstemmer library, which implements Porter's word stemming algorithm for collapsing words to a common root to aid in the comparison of vocabulary, the second one builds the word clouds, and the third one chooses the words’ colours. The wordcloud® function was parametrized to show all the words appearing at least 10 times in the corpus, with 35% of them vertically for good readability. A qualitative palette for qualitative data was chosen with the RColorBrewer® package, with which the sizes and colours of the words were defined according to their frequencies.

#### Selecting the health-related variables

To show convergence between CN-extracted data, socio-demographic and EMR data and how they enhanced residents’ health information, residents’ ages, entry ages, genders, medical histories and pathologies were added, as well as the number of falls and their severities, i.e., whether the physician was called or whether the resident was hospitalized [7, 26–28] The whole idea here was to build residents’ health features from health variables already in the database but not designed for this purpose (right side of Fig. 1). These variables were chosen as describing relevant residents’ features and emphasizing the problems afflicting the residents, especially those following physiotherapy programmes [28, 29]. The residents’ medical histories and pathologies followed the Pathos model [30] designed by the National Health Insurance Fund and the National Union of Clinical Geriatrics to assess the NH caregiving work load. Pathos is defined as a thesaurus of 50 pathological states, classified into ten domains: cardio-vascular, neuro-psychiatry, pleuro-pulmonary, infections, dermatology, osteo-articular, digestive, endocrine, uro-nephrology, and others (see Table 2). In fact, Pathos is a tool used by all NHs in France and it measures the residents’ care level needs through 8 resource posts: physician, psychiatrist, nurse, physiotherapy care, psychotherapy, biology, imaging and prescriptions. All these variables were well represented in the IS. Finally, the geographic level was integrated because medical histories or pathologies might differ depending where the residents lived. It was the same for the NH identification because physiotherapy care or its provision might differ in its description from one NH to another.

**Table 2:** The residents’ Pathos variable frequencies during their NH stays on 09/30/2013

Number of medical histories per resident as of 09/30/2013	0	1	2	3	4	5	6	7
*cardio-vascular*	*607*	*211*	*125*	*55*	*13*	*4*		
*neuro-psychiatry*	*571*	*205*	*117*	*66*	*32*	*16*	*7*	*1*
pleuro-pulmonary	896	111	8					
infections	941	69	5					
dermatology	954	58	3					
*osteo-articular*	*655*	*232*	*92*	*31*	*4*	*1*		
*digestive*	*709*	*200*	*80*	*26*				
endocrine	888	116	10	1				
uro-nephrology	873	119	22	1				
*others*	*679*	*235*	*71*	*27*	*3*			
Number of pathologies per resident as of 09/30/2013	0	1	2	3	4	5	6	7
*cardio-vascular*	*417*	*319*	*191*	*68*	*16*	*4*		
*neuro-psychiatry*	*290*	*291*	*230*	*125*	*66*	*9*	*4*	
pleuro-pulmonary	881	131	2	1				
infections	977	37	1					
dermatology	924	87	4					
*osteo-articular*	*622*	*279*	*89*	*20*	*4*	*1*		
*digestive*	*635*	*258*	*98*	*22*	*2*			
*endocrine*	*794*	*201*	*20*					
*uro-nephrology*	*758*	*231*	*25*	*1*				
*others*	*573*	*294*	*110*	*35*	*3*			

In italics all medical histories or pathologies with at least 200 residents (19.7%) afflicted at least once

#### Matching the physiotherapy residents’ narratives with their health-related subgroups through supervised classification

**Fig. 2:**
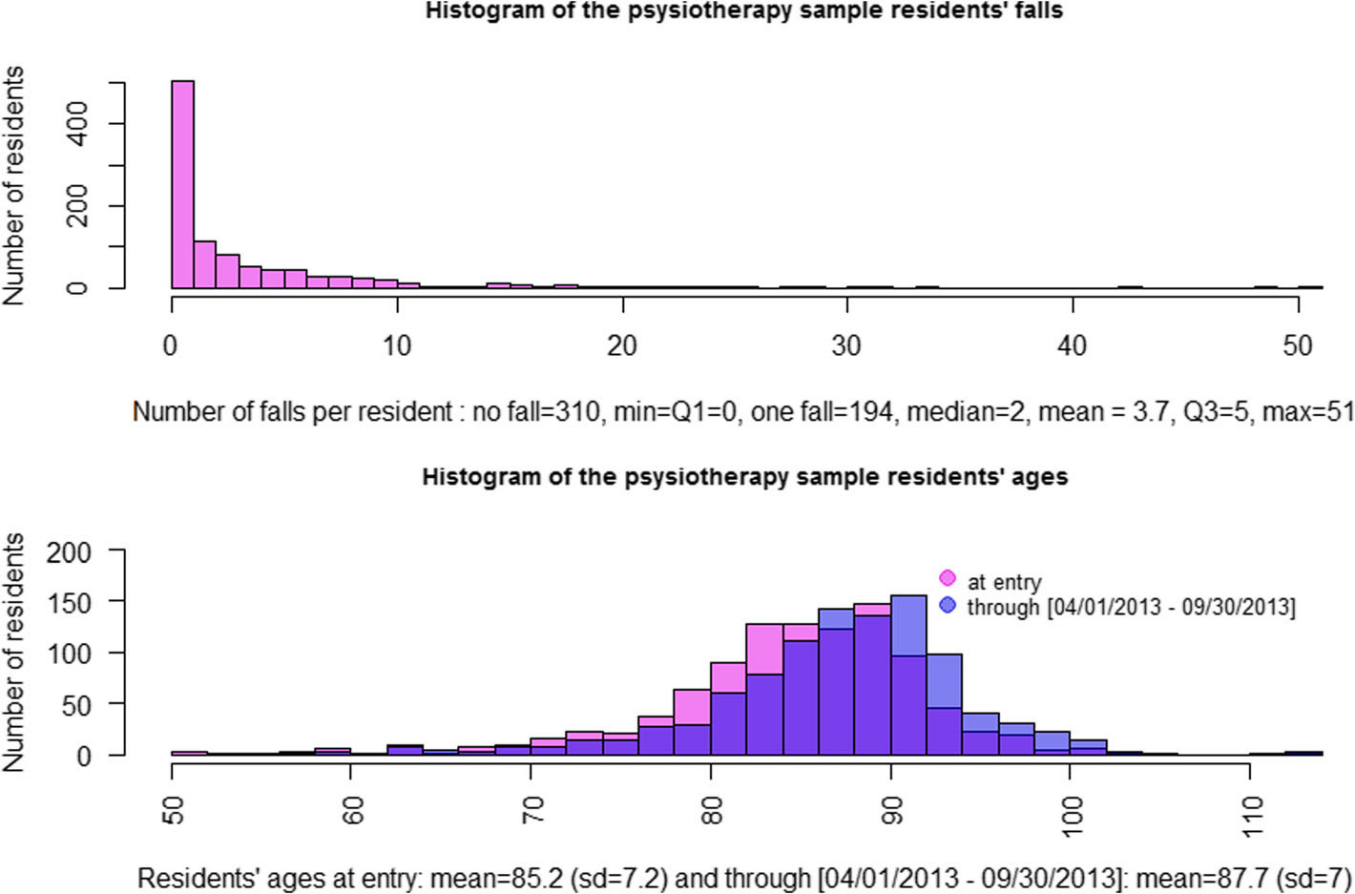
The residents’ number of falls and ages frequencies of the physiotherapy sample

Having built the residents’ health subgroups, we could now match the textual data with the health-related data (Fig. 1) to determine whether different health situations could be reflected by a variation in the vocabulary used. The two selected subgroups were a ‘falling’ group, including residents who fell at least 15 times since their NH entry, corresponding to the extreme number of falls’ class (see the Number of falls histogram in Fig. 2), and an ‘acute’ group, including residents who fell, and there was either a physician exam or a hospitalization following the fall. The two corresponding physiotherapy corpuses could then be built [7, 26, 27] using the 2 textual analysis stages discussed above, and they were compared qualitatively (the supervised health related subgroups analysed through text mining in Fig. 1 and detailed in Table 2).

#### Unsupervised data mining techniques to the residents’ health data

Subsequently, three unsupervised techniques were applied to the residents’ health data, principal component analysis (PCA) [31, 32], multiple component analysis (MCA) [33–35] and hierarchical clustering (HC) on principal components (PC) with the HCPC function [36, 37] of the R® package FactoMineR® [37], to determine whether the current data knowledge could be improved and helped by building physiotherapy health subgroups.

All three methods were used as dimension reduction techniques. PCA transforms the original variables into independent linear combinations of them. While PCA processes quantitative data using mostly Euclidian distance, MCA uses qualitative data and the Ch2 distance.

After the reduction step, scree plots in PCA and factor analysis are used to visually assess the components or factors that explain most of the variability in the data. Given the percentage of variation to be captured in the abridged data set, one could select the number of principal components to be considered.

For PCA, as for MCA, only the initial dimension can be retained to stabilize the clustering by deleting the noise from the data, which is essentially the HCPC function’s job. This function allows for the performing of HC and partitioning of the PC of several methods, choosing the best number of clusters and visualizing the tree, the partition and the principal components.

To apply MCA to our data, the residents’ ages, entry ages and numbers of falls were transformed into categorical variables. The HC on the MCA then proposed optimal cutting with 6 clusters (we can see below in Fig. 5 a strong inertia gain between five groups and six groups). Finally, the HCPC function [36, 37] was used to automatically retrieve every resident’s cluster number and identify his or her health’s characteristics.

## Results

There were 1015 residents (796 women and 219 men) entering NHs at a mean age of 85 years and 2 months old, staying there for an average time of 2 years and a half as of September 30, 2013, and falling an average of 3.7 times since their NH entry (see Fig. 2). The corpus of 4051 physiotherapy CNs provided an average of 4 CNs per resident for the six-month period, describing essentially physiotherapy care. For example, the list of words or short expressions appearing at least 5 times included: stopping_treatment, modifying_treatment, walking, autonomy, balance, cognition, per_day, per_week, antalgic, pain, renewal, functional_recovery, partial, motivation, voluntary, massage, stimulation, participation, and others. After cutting the CN at the 300th character (see above in the Building the physiotherapy variables subsection), the physiotherapy thesaurus was composed of 2165 different words with a mean length of 8.2 characters per word. Most of the CNs were composed of only one word (1588 CNs of 4051, 39.3%, with the first quartile Q1 = one word, Median = two words, Mean = 3.5 words, third quartile Q3 = 3 words, Maximum = 40 words).

### Characterizing the physiotherapy treatments through a small list of constructed key-words

**Fig. 3:**
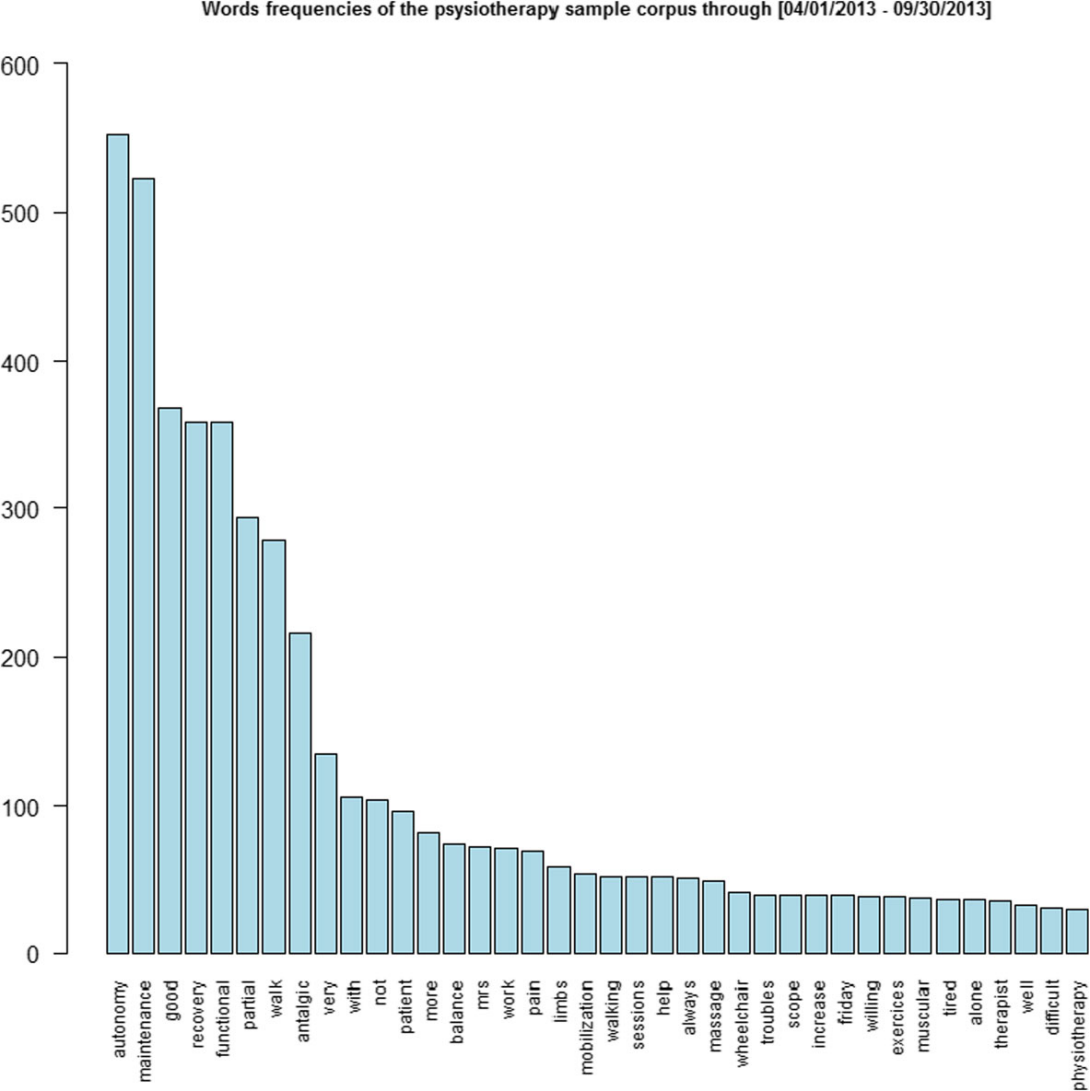
Bar plot of words appearing at least 30 times in the physiotherapy sample corpus (stage 2)

**Fig. 4:**
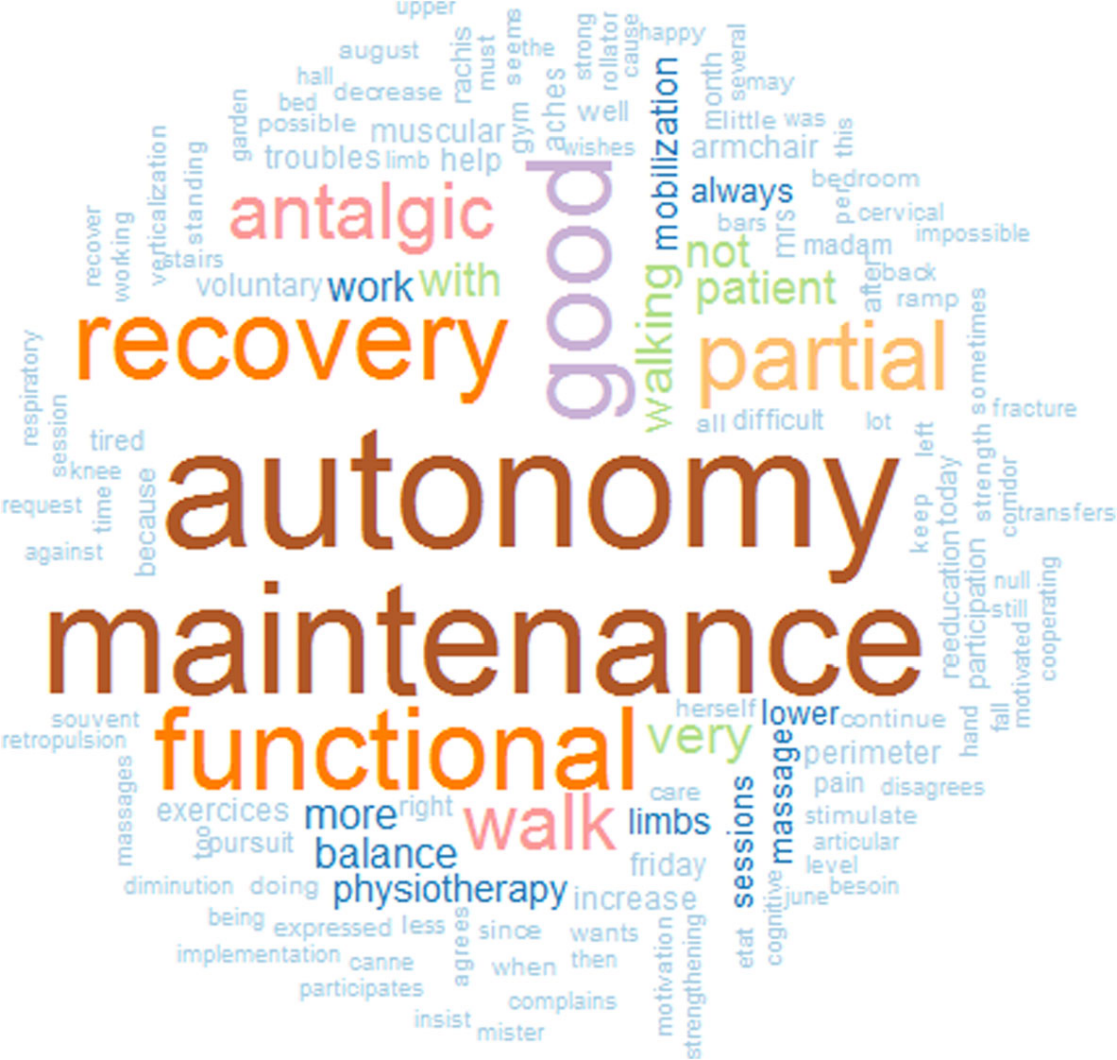
Word cloud with words appearing at least 10 times in the physiotherapy sample corpus (stage 2)

The two-stage process described above yielded the following results (Table 1 for the first stage and Figs. 3 and 4 for the second stage).

In Table 1, free_text observations (in italic) were those with more than 30 characters, and non_classified observations were those not containing any of the classified words listed above (in grey and black, respectively). When frequency numbers were followed by another number, the second ones stood for the number of times when they were used alone.

Between the first and second stages, the textual observations containing only the words ‘renewal’, ‘n (or) m times per_week’, ‘n (or) m times per_day’, ‘others’, ‘stopping_treatment’, and ‘modification_treatment’ were removed as observations not describing physiotherapy care, totalling 1427 observations (see Fig. 1), i.e., 35.23% of the corpus of 4051 observations. Hopefully, as said in building the physiotherapy variables subsection, we had a majority of one physio keyword - one physio concept expressions to describe the physio sessions and could then compute, except for *free_text* and *non_classified*, their precision as detailed here.

In the other cases, words used in sentences were most of the time rightly analysed. For example with the autonomy concept, we excluded the negative cases: *‘autonomy loss’*, *‘significant loss of autonomy’*, *‘resident who lost her autonomy in a wheelchair’*, idem for the pain concept: ‘*good mobility without pain’*, *‘not painful’*, *‘without pain’*; for the good concept we removed these three cases: *‘monitor the good device installation’*, ‘*hinder the smooth functioning of’*, ‘*it would be good to convince her’.* The real precision can be found on the last column table.

The bar plot in Fig. 3 lists the words appearing at least 30 times in the physiotherapy corpus. The first words were autonomy and maintenance, functional, recovery, good, partial, walking, and antalgic, as well as other words, such as work, limbs, mobilization and wheelchair.

The word cloud in Fig. 4 shows the words appearing at least 10 times in the physiotherapy corpus. Coming at the forefront were the same words -- autonomy and maintenance, functional and recovery, good, partial, antalgic and walk -- but also other words, such as anger, tired, recover and ache. Whereas the bar plot provides the precise word frequencies, the word cloud focuses on the residents’ difficulties and the therapist’s hard work with them.

### The residents’ health features

As detailed in the health-related variables selection subsection, gathering data from other files, the health residents’ features were described according to the Pathos model and were classified into ten domains (Table 2) plus their number of falls (Fig. 2), as well as their sex and age.

Any resident could have a medical history at entry or any pathology at all on 09/30/2013 (first column) up to six or seven of them (6th or 7th columns) in up to ten domains (cardio-vascular, neuro-psychiatry, etc.). Residents entered NHs mostly with cardio-vascular, neuro-psychiatric, articular and digestive problems, which usually increased after some time (in italics all medical histories or pathologies with at least 200 residents (19.7%) afflicted at least once). 

### Selecting two different health-related subgroups of residents through supervised classification and comparing their physiotherapy corpuses

Text mining of two residents’ health-related subgroups, built through supervised classification, found that even small differences in health situations resulted in qualitative and quantitative differences in physiotherapy narratives: 261 residents in the acute group and 58 in the falling group with, respectively, 993 and 232 textual observations. After removing the textual observations containing only the words ‘renewal’, ‘per_week’, ‘per_day’, ‘stopping_treatment’, and ‘modification_treatment’, (see above), there were still 513 and 109 textual observations containing 921 and 427 distinct words (Table 3), respectively.

**Table 3:** The word frequencies of the acute and falling groups’ physiotherapy corpuses computed with tm®

Hospitalized or followed by a physician after falling (261 residents)			Fallen at least 15 times (58 residents)		
word	frequency	ratio	word	frequency	ratio
autonomy	127	0.49	autonomy	29	0.50
functional	97	0.37	walking	23	0.40
recovery	96	0.37	functional	16	0.28
walking	64	0.25	recovery	16	0.28
antalgic	45	0.17	mobilization	12	0.21
very	42	0.16	very	11	0.19
plus	34	0.13	help	10	0.17
patient	28	0.11	plus	10	0.17
work	26	0.10	always	9	0.16
always	19	0.07	work	8	0.14
limbs	18	0.07	massage	7	0.12
massage	17	0.07	physiotherapist	6	0.10
mobilization	16	0.06	limbs	6	0.10
troubles	15	0.06	going further	6	0.10
help	13	0.05	rachis	6	0.10
pain	13	0.05	re-education	6	0.10
exercises	13	0.05	good	5	0.09
sessions	13	0.05	wheelchair	5	0.09
friday	13	0.05	fracture	5	0.09
good	12	0.05	less	5	0.09
difficult	12	0.05	new	5	0.09

In this table, each word frequency is followed by its sample ratio. For example, the word ‘autonomy’ appeared 127 times in the acute group and 29 times in the falling group. Words’ ratios reflect the physiotherapy care priorities, which can differ in each group. For example, functional and recovery seem more meaningful in the acute group (37%) than in the falling group (28%).

### Unsupervised health related subgroups built through data-mining techniques

**Fig. 5:**
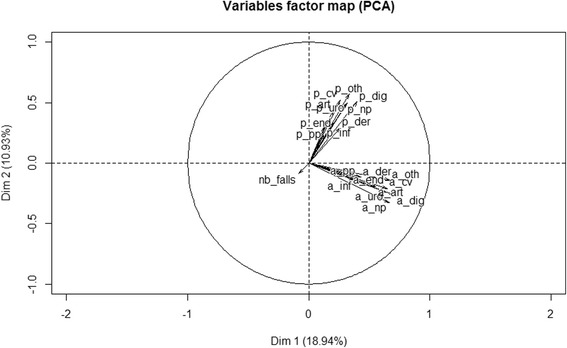
The PCA on medical histories, pathologies and number of falls defined as continuous variables

As explained in the unsupervised data mining techniques on the residents’ health data subsection, exploratory techniques, PCA and MCA, followed by clustering on these data, were used to visualize different subgroups with different needs. First, PCA was performed because our variables were essentially numeric (except for the NH names, the regions and the sex, not studied here) (see Fig. 5).

The eigenvalue decomposition yielded seven eigenvalues greater than one, but together, they defined only 59% of the total inertia, with the two first principal components bringing less than 30% of it (18.94% + 10.93% giving 29.87% see Fig. 5). Plus, all medical histories (variables a_xx) followed one direction and all pathologies (variables p_xx) another perpendicular to the first one, indicating that the health problems at the residents’ NH entry were mostly ‘independent’ of their heath situations after a while. Finally, the number of falls (variable nb_falls) pointed to a third direction perpendicular to the first principal plan.

**Fig. 6:**
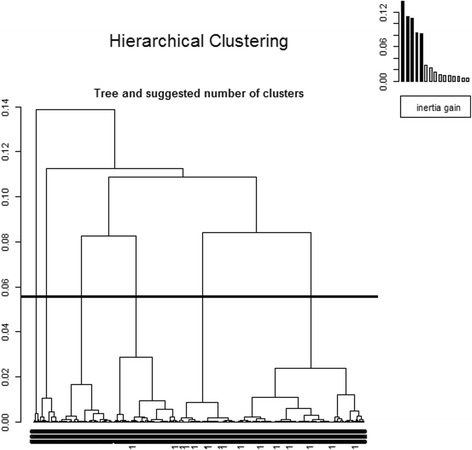
HC + MCA with regions, departments, NH’s names, gender, medical histories and pathologies as categorical variables

**Fig. 7:**
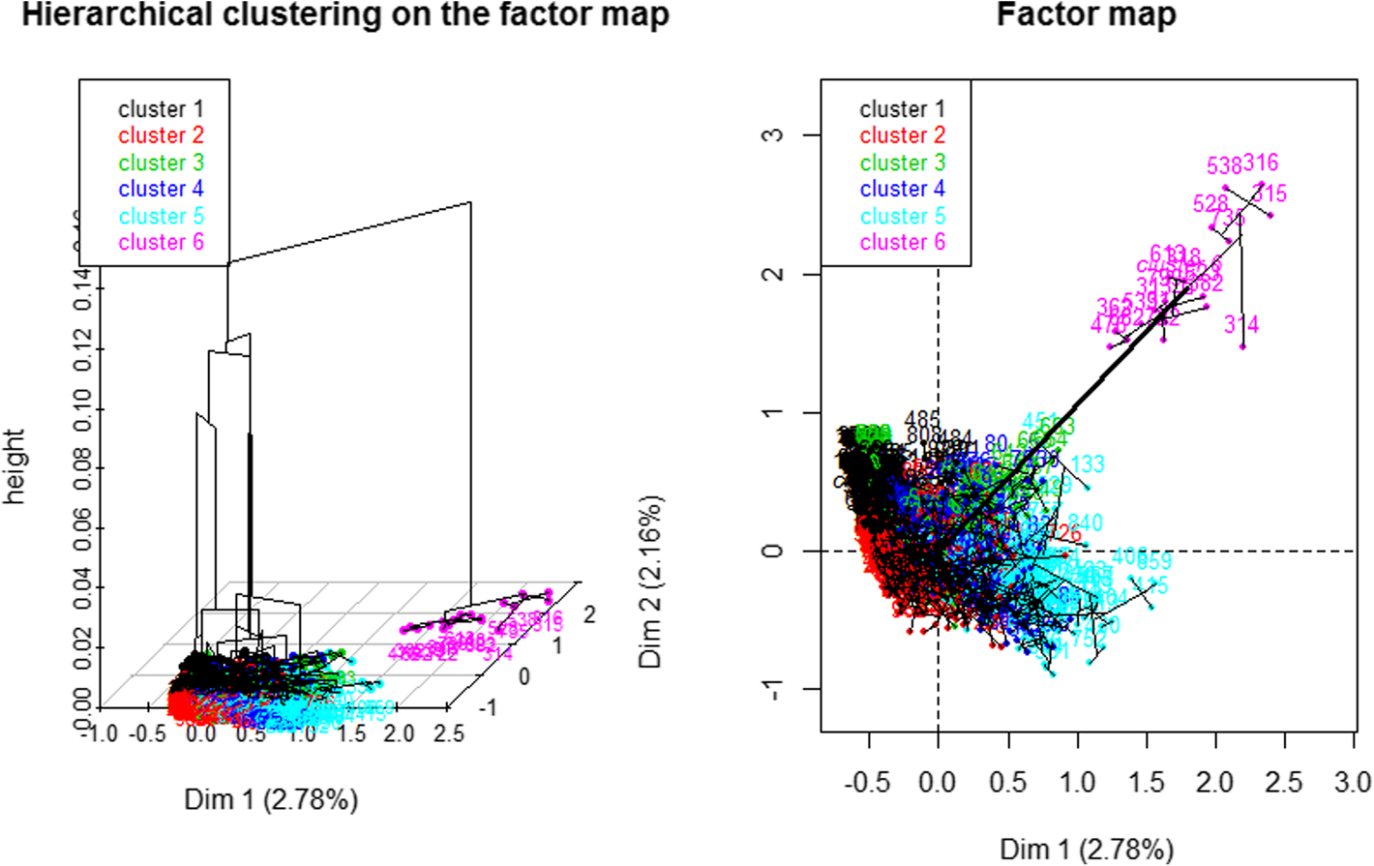
The HCPC plot function of the 6 clusters in 3D and 2D

Then MCA was performed, adding the NH names, regions and departments, keeping at first the ages and number of falls as illustrative variables, after casting the numbers of medical histories and pathologies as categorical variables, and using the HCPC function. The HC on the MCA proposed an optimal cutting with 6 clusters (see in Fig. 6 the horizontal line showing the best clustering cut, plus a strong inertia gain between five and six clusters), which why, as explained in the unsupervised data mining techniques on the residents’ health data subsection, the three continuous variables -- age, age at the NH entry and number of falls -- were also divided into six-quantile groups to cast them into qualitative variables, using cut-off points as fairly as possible to follow the best clustering number based on the HCPC function, hoping to obtain the most discriminant residents’ partition. All of these categorical variables were strongly discriminatory factors, especially the geographic variables, contributing most significantly to the global inertia (for example, the Chi2-test yielded, for the NH names, region and department variables, 3 null *p*-values). The HCPC plot function in 3D and 2D with colours showed 6 clusters (Fig. 7) with the HCPC modelling of the last variable providing the cluster number. There were 229 residents for the first one, 335 for the second, 58 for the third, 208 for the fourth, 147 for the fifth and *n* 1 for the last one, which was coloured in magenta lying far from the five others. After examining the sixth cluster’s residents, all of the residents, except one, had no pathology at all but numerous medical histories. Furthermore, they all came from the same NH. One last MCA plus clustering was attempted, adding age, age at NH entry and number of falls, defined as categorical variables, with 6 modalities, which then yielded five clusters instead of six, the fifth corresponding to the sixth one before, and the five clusters becoming four.

Finally, by removing these *n*, all the remaining residents were still near the gravity centre.

## Discussion

Through this textual experiment, it was shown that text mining and data mining techniques could add new, simple, useable data to EHR to improve residents’ health and quality of care.

To achieve this goal and because only limited studies of strategies designed to increase the efficiency of processing CNs exist [12], it was decided to work on these textual data [14] to understand their characteristics through data mining, which is the core technology of customer relationship management [38] and one of the best tools to analyse the quality of care in this type of data frame. This physiotherapy CN sample might contain many specialized medical terms with no normalization expected, but it was believed that this textual information, which was potentially available, might be transformed into useful and understandable knowledge to facilitate professional practice and active interdisciplinary collaboration and research [39].

This textual experiment with the physiotherapy treatments, using a textual process in two stages, displayed convenient tools to improve the residents’ health and quality of care by adding new, simple, useable data to the EHR. Text mining and data mining techniques on this DWH enriched the residents’ health features by adding new, simple, useable data, such as physiotherapy keyword frequencies, to the EHR, opening the way towards more substantial textual information uses, such as patient stratification and improved targeting of care [15].

This textual approach also gives new meaning to all the time spent by medical assistants, nurses and doctors feeding the residents’ EHR without changing their working habits or adding new tedious tasks.

The textual process in two steps, as presented in Table 1 (first step with SQL) and Fig. 3 (second step with tm®), offered better control of the quantitative and qualitative aspects of the textual analysis: the corpus analysed by tm® was already cleaned up of frequent and meaningless words, and conversely, meaningful words or expressions appeared at the forefront. This method in two steps attempted to resolve the research issue of detecting truly meaningful words in this corpus, as is usually done with the tf-idf technique with its word weighting scheme, which helps to adjust for the most frequently occurring terms often not being the most meaningful ones, especially for the user [40].

Shortening the physiotherapy care narratives from 4000 characters to 300, as there was a majority of short character fields, allowed us to expedite the textual extractions and ease the textual analysis [3]. Examining the difference when selecting the first 400 characters, instead of the first 300, we found only 34 CNs, less than 1% longer than 300 char, and we compared the two sets’ 45 most frequent words to find no significant difference between the two sets (see Additional file 5: Annex 5). Other strategies with NLP methods to increase efficiency and usefulness, such as extracting concepts or filtering high word counts, can be found in [12, 17].

Figure 3 shows that few terms were used frequently, such as autonomy, maintenance, recovery or functional (the left portion of Fig. 3), while many terms were used infrequently, such as exercises, troubles, re-education, difficult, pain or participation (the bottom/right portion) [17]. This finding is reminiscent of Zipf’s law [41], which describes the empirical frequency distribution of words in general language as having a large peak and a heavy one-sided tail. As in [17], the most frequent terms were general rather than specific and reflected the domain from which they arose.

This study had several strengths. First, by comparing the numbers of keywords and expressions of both steps, the tm process could be checked, and the whole text mining process could be iteratively improved to provide a detailed description of the physiotherapy sessions and treatments. Even after choosing to stop at the second step, it remained possible to refine the SQL queries gradually, adding new expressions and using wildcards to extract more complex textual data, as will be done in the future. Additionally, by working like this, a physiotherapy care keyword list was defined, which could be enriched and improved. It worked like a problem list, as was done in the UMLS-CORE project [42, 17]. This technique will allow us to reuse it later with other IS in our group and ease their overlapping. Also by design and as shown in Table 1, this list was built using simple SQL queries. In each query, what was searched for was precisely what was identified: for instance, the word balance in the physio CN described with an almost 100% precision what these physio-sessions were all about: improving the resident’s balance and nothing else (except for one case with balance, *‘unaware of his weak balance’*). It was mostly the case for walking (improve walking), pain (alleviate pain), massage, motivation and so on. Finally, much better visualization of the words describing physiotherapy care was available, with this *‘filtered’* word cloud, which is good at communicating qualitative data and can be a simple tool to identify the focus of written material [43] as well as capture risk perception [44].

Second, by selecting medical history, pathologies and number of falls and by adding the regions, departments and NH identifications, it became possible to describe all of the residents and their NH locations following physiotherapy sessions over a six-month period and to detect some data discrepancies.

By performing PCA of the residents’ health groups, one interesting result was found: all of the medical histories followed one direction and all of the pathologies another, perpendicular to the first, suggesting that the residents’ health groups changed greatly over time. However, the number of falls pointed to a third direction perpendicular to the first principal plan, while being one of the main risk factor in which we were interested [26, 7, 27]. Nevertheless, by checking with another PCA method using the built-in R stats® package prcomp function [45], adding this time age and entry age, we found even less inertia (27%), with the first two PCs defined as before by medical histories and pathologies and the third one being essentially defined by age and entry age. With MCA, rather than PCA, followed by HC, we found why one of the clusters was so far from the other five: in MCA, 2 individuals are nearer if they have common rare modalities for one or more variables: here all ten p_xx variables, standing for pathologies, had a zero value for all except one resident of the last cluster. Looking further at Fig. 7, the distance between the 6th cluster and the five others might have come essentially from the absence or presence of pathologies. It is likely that, because medical histories and pathologies are fed into the same table, there was an omission or oversight when feeding the database. However, this group was also found to be older (1 year and a half) and to have fallen more (2 times more). Finally, by removing these *n* residents, all of the remaining were still near the gravity centre as before, and we concluded that this sample was very homogeneous with our criteria: age, entry age, sex, medical histories and pathologies in 10 domains, gravity and number of falls. With more heterogeneity in the data, it might have been possible to find more disjointed health subgroups and interesting differences to describe.

Third, using text mining [22] on two slightly different residents’ samples showed that even small differences in health situations yielded to easy to detect qualitative and quantitative differences in physiotherapy narratives (Table 3). Autonomy was the main objective in both groups, as it was for the whole sample (see the words *autonomy maintenance* in Figs. 3 and 4) in, respectively, 49% and 50% of physiotherapy care narratives; then, the words functional recovery (37% as seen in Figs. 3 and 4) for the acute group versus walking (40% idem) for the falling group; and finally, the words antalgic (17% idem) and pain (5% idem) in the acute group and not in the falling group.

Nevertheless, this study had several limitations. First, it was observational and explored only a selected sample of this CN database on a restricted subject, physiotherapy comments, and with a short follow-up period of 6 months, but throughout this whole experiment, we could better know the free text content of our CN and how to use it by building dummy variables defining the physiotherapy treatment and checking their values. Second, while word stemming was used when performing the SQL queries with the LIKE function and wildcards [21], this option was not selected when using package tm® for two reasons. First, it did not work well in French; for example, even if it cut the word *marche* (walking) into *march*, the words *marche* and *marcher* (to walk) were not combined. It was the same thing for the words *bon (good)* and *bonnes (good)*: *bonnes* was cut into *bonn* but was still different from *bon* and so was counted as another word. Second, when the words are mapped into word clouds, it is more difficult to read the words’ stems than the words. We attempted to define the best trade-off between data visualization and accuracy of the data.

Finally, matching residents with their CNs relied on physiotherapy care observations, and physiotherapy care discrepancies were found between NHs. For example, physiotherapy care descriptions varied greatly in frequency from one region to another: there were 201 physiotherapy observations for the North-West region with 17 NHs, whereas there were 754 of them for the South region with the same number of NHs, showing that, without normalization, the textual data are highly dependent on the style, precision and depth of physiotherapy care descriptions and the people feeding the IS. Using here a normalized physiotherapy problem list systematically for every physiotherapy session could help to solve this problem, but even without normalization, additional information is often available in unstructured free text, as shown with the experiment in the UK with rheumatoid arthritis (RA) in primary care of the general population [46, 47].

## Conclusion

Through text and data mining techniques, an empirical approach to integrating health narratives into the existing information system was illustrated. Thanks to this physiotherapy data textual analysis in two stages (Fig. 1), new health variables describing residents’ autonomy, functional recovery and pain or walking difficulties were built. This textual data could help define health subgroups, such as at-risk or recurrent fallers, through classification or could predict future health problems, such as hospitalizations or deaths, through logistic regression machine learning algorithms.

These new semantic technologies could improve the residents’ follow-up over time and their health paths and could offer well-adjusted solutions to their multiple health problems. As with incentive programmes of the Centers for Medicare and Medicaid Services in the United States [48], it should be possible to answer questions about the meaningful use of EHR and quality of health care in the near future.

As was just said, combining structured data (age, gender, health groups) and unstructured data (physiotherapy care narratives) together provided a richer resident description [49]. MCA plus clustering could help differentiate residents, for example, those not having any pathologies, and could enable greater patient stratification, for example, through NH indexation or regions.

Nevertheless, to be truly useful, all of these health observations must be normalized to be reused later or be compared with other data samples, qualitatively and quantitatively. As explained in the Handbook on Research on ICT (Information and Communication Technology) for Human-Centered Healthcare and Social Care Services [50], the critical bottleneck today is, namely, information handover and reuse, and only interactively validated and semantically processed texts can be helpful to all health parties. To achieve this goal, problem lists must be defined for every type of health domain, as has been done in another DWH of the (Korian) group. There, health problem lists are in one table and the narratives in another one, pointing to the first one through indices. Another experiment in the UK used a complex comprehensive process of developing code lists building the clinical entity RA (rheumatoid arthritis) [46] through indicators and markers, and a third one, in the Boston area [51, 52], examined clinician and health care providers’ attitudes towards problem lists in EHR and found that a common approach, completeness and standardization were necessary.

We showed here that CN could add valuable health information to the residents’ health data in our database. We are confident in using it further through a clinician’s finely tuned keywords and problems list, describing many geriatric ailments in the whole resident population, as was done for RA in the UK general adult population [46].

As explained in [52], future work on CN data should optimize the use of key functions to improve health providers’ time efficiency, as well as data quality, integrity and usefulness [48, 53]. The next step will then be to fully integrate the CN data, with the problem list being the main tool defining health key functions. This goal will be met with finely tuned health data textual extraction analysing the whole textual generation process and relying on real health data content and staff uses. We hope being able to improve preventive health by better characterizing residents’ falls following influenza vaccinations as well as through better management of chronic diseases, such as cancer, chronic pain, diabetes or dementia.

## Additional files

Additional file1: Empirical Advances with, Annex 1, The physiotherapy corpus, content: the 2616 remaining de-identified textual clinical narratives of the 1015 residents’ sample in French (XLS 233 kb)

Additional file 2: Empirical Advances with, Annex 2, The physiotherapy corpus anonymization, content: describes the physiotherapy corpus de-identification and anonymization process (DOC 39 kb)

Additional file 3: Empirical Advances with, Annex 3, The physiotherapy corpus translation, content: describes the physiotherapy corpus translation process from French to English. (DOC 26 kb)

Additional file 4b: Empirical Advances with, Annex 4, The 1015 residents’ health table, content: the 1015 de-identified residents’ medical histories and pathologies on September 30th 2013, as well as their falling history and 10-anonymized NHs (XLS 256 kb)

Additional file 5: Empirical Advances with, Annex 5, Comparing the two sets of key words with 300 char and 400 char, content: compares the physiotherapy narratives’ corpuses cut after 300 characters and after 400, in fact the most frequent words in the two corpuses, with a Chi2 test. (DOC 48 kb)

## Abbreviations

CN: Clinical narrative
DM: Data mining
DWH: Data warehouse
EHR: Electronic health record
EMR: Electronic medical record
HCPC: Hierarchical clustering on principal components
IE: Information extraction
IS: Information system
MCA: Multiple correspondence analysis
NER: Named entry recognition
NH: Nursing home
PCA: Principal components analysis
RA: Rheumatoid arthritis
SQL: Standard Query Language

## Footnotes

1
*n* < 30. We removed the real number for NH de-identification and for data harmonization.
