# Supplementary material for: Empirical advances with text mining of electronic health records
Source: BMC Med Inform Decis Mak. 2017 Aug 22;17:127. doi: 10.1186/s12911-017-0519-0 (PMC5568397; doi:10.1186/s12911-017-0519-0)
Supplement: Supplementary file 2 — Empirical Advances with, Annex 2, The physiotherapy corpus anonymization, content: describes the physiotherapy corpus de-identification and anonymization process (DOC 39 kb) [file 12911_2017_519_MOESM2_ESM.doc]

**The physiotherapy corpus de-identification**

We did the de-identification, the codification of residents’ and doctors’ names but with the retention of some identifying information [10] just after the physiotherapy selection process (see figure 1). To do this we proceeded in two steps.

First, we searched all the ‘*MR’*, ‘*MONSIEUR’ (mister), ‘M. ‘, ‘M ‘,* and changed them into *‘MR_’*. We did the same for all the *‘MM’*, ‘*MADAME’ (madam)*, *‘ME’* and changed them into *‘MM_’*. Then we added sequentially defined suffixes keeping track of every resident in the whole physiotherapy corpus. We used the same technique for the doctors: we searched all the ‘*DR’*, ‘*DOCTEUR’ (doctor), ‘ORDO’ (prescription)* and changed them into *‘DR_’* adding sequentially defined suffixes keeping track of every doctor in the whole physiotherapy corpus.

Second, we re-indexed all the residents’ index in order to protect personal privacy but be able to re-link them later by a trusted party if later needed. The new indexes were computed through a piecewise linear increasing function smoothed on the original indexes. Without an access on the Korian group database it is then nearly impossible to find the matching between old and new indexes.

**The 1015 health profiles’** **anonymization and de-identification**

We re-indexed all the residents’ health profiles and just anonymized the NH names by renaming all the nursing homes as ‘KORIAN_’ with sequentially defined suffixes, based on the initial residents’ sorting plus whenever there were less than 10 CN per NH, we removed the region and department names to sever irreversibly NH and residents’ identities to prevent any future re-identification [17-19]. We also deleted age, age at entry and sex following the editor’s counsel. Even with 23 sensible fields as medical histories, health and risk factors (see table 3), all the residents’ health and risk factors frequencies were computed through a whole query process and are not readily accessible. Plus medical histories, health and risk factors could take at least three different values. With all these tools we built a 10-anonymised, 3-diversified sample and kept all sensible information except the geographic information for small residents’ subsets.
