# Supplementary material for: Empirical advances with text mining of electronic health records
Source: BMC Med Inform Decis Mak. 2017 Aug 22;17:127. doi: 10.1186/s12911-017-0519-0 (PMC5568397; doi:10.1186/s12911-017-0519-0)
Supplement: Supplementary file 3 — Empirical Advances with, Annex 3, The physiotherapy corpus translation, content: describes the physiotherapy corpus translation process from French to English. (DOC 26 kb) [file 12911_2017_519_MOESM3_ESM.doc]

**The physiotherapy corpus translation**

We did the translation of the physiotherapy corpus at the end of step 1 (see figure 1). We checked the most frequent keywords and specific physiotherapy expressions of the corpus and translated them all at once. For example ‘*maintenance de l’autonomie’* and ‘*récupération fonctionnelle’* were translated as ‘*autonomy maintenance’* and ‘*functional recovery’* all at once. Then, for the remaining most frequent expressions or words, we translated them one by one to keep the real meaning. For example the word ‘*pas’* was translated either as ‘*not’* or as ‘*step(s)’*, depending on the context, taking ‘*not’*  into account and not as a French stop word. That’s why the word not can be found both in figures 2 and 3. Plus, we kept orthographic and syntax mistakes, not translating them to the expected expressions. By working that way, we tried not improving the corpus which stayed as in real life and hoped not introducing some bias. So that’s why we can find some discrepancies between the ranks of some keywords of the figure 2 bar plot (translated in English and then ordered) and those ordered in French and then translated.
