# Supplementary material for: Empirical advances with text mining of electronic health records
Source: BMC Med Inform Decis Mak. 2017 Aug 22;17:127. doi: 10.1186/s12911-017-0519-0 (PMC5568397; doi:10.1186/s12911-017-0519-0)
Supplement: Supplementary file 5 — Empirical Advances with, Annex 5, Comparing the two sets of key words with 300 char and 400 char, content: compares the physiotherapy narratives’ corpuses cut after 300 characters and after 400, in fact the most frequent words in the two corpuses, with a Chi2 test. (DOC 48 kb) [file 12911_2017_519_MOESM5_ESM.doc]

**Comparing the two sets of key words with 300 char and 400 char**

We extracted the words appearing at least 25 times in the physiotherapy care narratives (see step 2 of figure 1), shortened to the first 300 char and then to the first 400 char and found the following results.

| **first 300 char** | **first 400 car** |
| --- | --- |
| **freq ratio**  maintenance 530 0.52  autonomy 553 0.54  recovery 366 0.36  functional 359 0.35  good 366 0.36  partial 294 0.29  walk 243 0.24  antalgic 216 0.21  very 133 0.13  more 81 0.08  patient 73 0.07  work 64 0.06  balance 63 0.06  physiotherapy 59 0.06  limbs 59 0.06  mobilization 54 0.05  walking 53 0.05  sessions 51 0.05  always 51 0.05  massage 49 0.05  armchair 41 0.04  aches 40 0.04  mrs 40 0.04  perimeter 40 0.04  Friday 39 0.04  exercices 38 0.04  troubles 38 0.04  voluntary 38 0.04  increase 37 0.04  well 32 0.03  madam 32 0.03  muscular 30 0.03  little 30 0.03  pain 29 0.03  reeducation 29 0.03  difficult 27 0.03  participation 27 0.03  pursuit 26 0.03  rachis 26 0.03  alone 26 0.03  help 25 0.02  strength 25 0.02 | **freq ratio**  maintenance 531 0.52  autonomy 554 0.55  recovery 366 0.36  functional 359 0.35  good 366 0.36  partial 294 0.29  walk 247 0.24  antalgic 216 0.21  very 135 0.13  more 82 0.08  patient 73 0.07  work 65 0.06  balance 63 0.06  physiotherapy 60 0.06  limbs 60 0.06  mobilization 54 0.05  walking 55 0.05  sessions 51 0.05  always 52 0.05  massage 49 0.05  armchair 44 0.04  aches 40 0.04  mrs 40 0.04  perimeter 41 0.04  Friday 39 0.04  exercises 41 0.04  troubles 38 0.04  voluntary 38 0.04  increase 39 0.04  well 33 0.03  madam 34 0.03  muscular 32 0.03  little 30 0.03  pain 29 0.03  reeducation 29 0.03  difficult 27 0.03  participation 28 0.03  pursuit 26 0.03  rachis 26 0.03  alone 28 0.03  help 27 0.03  strength 25 0.02 |

We compared the two samples with a Chi2 test and found no significant difference:

Pearson's Chi-squared test

X-squared = 0.6087, df = 44, p-value = 1
